# Comparative effectiveness of traditional Chinese medicine treatment strategies for insomnia: a systematic review and network meta-analysis

**DOI:** 10.3389/fneur.2026.1912776

**Published:** 2026-09-25

**Authors:** Yan Liu, Xiuying Wang, Yaqin Zhang, Yanfang Li, Yini Huang

**Affiliations:** Department of Neurology and Geriatrics, Changsha Hospital of Traditional Chinese Medicine (Changsha No. 8 Hospital), Changsha, Hunan, China

**Keywords:** insomnia, network meta-analysis, sleep disorder, traditional Chinese medicine formulations, Western medicine

## Abstract

**Objective:**

Insomnia causes daytime impairment and reduced quality of life. We evaluated the comparative efficacy and safety of Traditional Chinese Medicine (TCM) treatment strategies for insomnia.

**Methods:**

Nine databases (PubMed, Embase, Web of Science, Cochrane CENTRAL, ClinicalTrials.gov, CNKI, Wanfang, VIP, SinoMed) were searched through January 2026 for randomized controlled trials (RCTs). Primary outcomes were ISI and PSQI scores; secondary outcomes included clinical efficacy and adverse events (AEs). Data were synthesized using frequentist network meta-analysis.

**Results:**

Twelve RCTs involving 1,260 participants were included, evaluating five strategy nodes: TCM alone, TCM plus Western medicine (WM), WM alone, placebo, and CBT-I. For ISI, TCM plus WM ranked highest (SUCRA 89.7%) and significantly improved scores versus placebo (OR 2.84, 95% CI 1.42–5.68) and WM (OR 1.96, 95% CI 1.08–3.56). For PSQI, TCM plus WM showed the highest probability of improvement (SUCRA 71.9%) with greater reduction versus placebo (MD −2.31, 95% CI −3.65 to −0.97). For clinical efficacy, SUCRA values were 86.6% for TCM plus WM, 77.2% for TCM, 20.9% for WM, and 15.3% for placebo. For AEs (higher SUCRA indicating better safety), SUCRA values were 94.8% for WM, 43.9% for TCM plus WM, 31.1% for placebo, and 30.2% for TCM. Sensitivity analysis excluding secondary insomnia confirmed robust findings.

**Conclusion:**

Integrated TCM-WM and TCM therapies demonstrate clinical efficacy and acceptable safety for insomnia management.

**Systematic review registration:**

The review protocol was prospectively registered in the International Platform of Registered Systematic Review and Meta-analysis Protocols (INPLASY) (Registration number: INPLASY202680041; DOI: 10.37766/inplasy2026.8.0041).

## Introduction

Insomnia is one of the most common sleep disorders, characterized by difficulty initiating sleep, difficulty maintaining sleep, or early morning awakening, accompanied by significant daytime functional impairment, reduced quality of life, mental disorders, and substance use disorders (1, 2). The prevalence of insomnia is considerable, affecting approximately 35 to 50% of adults, with up to 20% meeting the diagnostic criteria for insomnia. Chronic insomnia is associated with depressive symptoms, anxiety, cognitive impairments such as memory and attention deficits, and poor occupational performance (3).

Existing treatments for insomnia include cognitive behavioral therapy and pharmacotherapy (4). The first-line treatment for persistent insomnia is typically Cognitive behavioral therapy for insomnia (CBT-I), which employs techniques such as relaxation, stimulus control, sleep hygiene education, and sleep restriction (5). Although CBT-I has been proven effective, its application is limited by a shortage of trained clinicians and high costs, factors contributing to a dropout rate as high as 34% (6, 7). Pharmacotherapy, including certain antidepressants, melatonin receptor agonists, non-benzodiazepine receptor agonists, and benzodiazepines, is commonly used for managing insomnia. However, these medications require cautious use due to potential adverse effects and are generally recommended only for short-term management (8). Widely used drugs may be associated with cognitive deficits, tolerance, and dependence, and the overall efficacy of insomnia medications remains controversial, with limited objective improvements observed in sleep quality or daytime function (9).

Traditional Chinese Medicine (TCM) provides a different perspective for the treatment of insomnia and focuses on physiological imbalances in the different organ systems inside the body and also stresses lifestyle changes (10). TCM is increasingly considered as a complementary therapeutic approach for insomnia, with some studies suggesting potentially favorable tolerability profiles. However, the long-term safety, risk of adverse effects, and comparative effectiveness of different TCM interventions require further rigorous evaluation. In clinical practice, various TCM therapies, including herbal formulas (e.g., Tianwang Buxin Dan, Suanzaoren Tang), Chinese patent medicines, acupuncture, tuina (therapeutic massage), and herbal baths, are widely applied in the management of insomnia, and substantial experience has been accumulated (11–13). In recent years, numerous systematic reviews and meta-analyses have also suggested the potential efficacy of TCM therapies in improving sleep quality, with reported potential benefits and generally acceptable short-term tolerability in available studies (14–16); however, the certainty of evidence remains variable because of limitations in trial quality and follow-up duration (14, 15).

Current research predominantly focuses on pairwise comparisons between a single TCM therapy and Western medicine or a vehicle, lacking direct and indirect comparisons among multiple TCM herbal formulas themselves, as well as with conventional treatment modalities. Furthermore, the relative advantages and disadvantages of different TCM interventions regarding clinical effective rate, degree of sleep quality improvement, and risk of adverse reactions remain unclear, which hinders the scientific and individualized nature of clinical decision-making. Network Meta-Analysis (NMA), as an advanced statistical method capable of simultaneously integrating both direct and indirect evidence, enables the ranking and comparison of multiple interventions, thereby providing an evidence-based foundation for selecting the optimal treatment plan in clinical practice (16).

In this study, the term “insomnia” was used as an umbrella term referring to clinically significant insomnia symptoms or insomnia disorders. Primary/chronic insomnia refers to insomnia occurring independently of other medical conditions, whereas secondary or comorbid insomnia refers to insomnia associated with neurological, psychiatric, oncological, or other medical conditions. Considering that insomnia symptoms frequently coexist with other disorders, trials were considered eligible when insomnia represented the primary therapeutic target and standardized sleep-related outcomes were evaluated. This approach allowed inclusion of clinically relevant insomnia populations while maintaining a clear focus on insomnia-related treatment effects.

Therefore, we aimed to perform a NMA to integrate evidence from randomized controlled trials (RCTs) evaluating TCM formulations for insomnia and to compare their relative efficacy and safety with conventional interventions. By synthesizing both direct and indirect evidence, this study sought to clarify the comparative effectiveness of different TCM-based therapeutic strategies, including TCM alone, TCM combined with Western medicine, Western medicine alone, placebo, and CBT-I. The main contributions of this study include: (1) comparing multiple TCM formulations and commonly used interventions using a network meta-analysis framework; (2) evaluating both efficacy-related outcomes and safety profiles across different treatment strategies; and (3) providing comparative evidence to support future clinical research and individualized treatment decisions for insomnia management.

## Protocol

This systematic review and network meta-analysis was conducted according to the Preferred Reporting Items for Systematic Reviews and Meta-Analyses (PRISMA 2020) (17) and PRISMA-NMA guidelines. The review protocol was prospectively registered in the International Platform of Registered Systematic Review and Meta-analysis Protocols (INPLASY) (Registration number: INPLASY202680041; DOI: 10.37766/inplasy2026.8.0041). Any methodological decisions were consistent with the registered protocol.

### Search strategy

Two researchers independently searched multiple electronic databases from inception to January 31, 2026, including PubMed, Embase, Web of Science Core Collection, Cochrane CENTRAL, ClinicalTrials.gov, CNKI, Wanfang Data, VIP Database, and SinoMed. Because a substantial proportion of TCM clinical trials are published in Chinese-language journals, both English and Chinese databases were searched to minimize language and publication bias. No language restrictions were applied. The search aimed to identify RCTs evaluating TCM formulations for insomnia.

The search strategy was developed using controlled vocabulary and free-text terms related to insomnia, TCM, and randomized controlled trials. The core search strategy was:

(insomnia OR insomnia disorder OR chronic insomnia OR sleep disturbance OR sleep initiation dysfunction) AND (Traditional Chinese Medicine OR Chinese herbal medicine OR herbal formula OR Chinese medicine OR TCM) AND (randomized controlled trial OR randomized trial OR clinical trial).

The complete search strategies for all databases, including PubMed, Embase, Web of Science, Cochrane CENTRAL, ClinicalTrials.gov, CNKI, Wanfang Data, VIP Database, and SinoMed, are provided in Supplementary material.

### Inclusion criteria

The primary objective of this study was to evaluate randomized controlled trials investigating TCM formulations for insomnia disorders. Eligible studies included adults diagnosed according to internationally or nationally recognized diagnostic criteria, including DSM-IV, DSM-5, ICSD criteria, ICD criteria, or validated Chinese diagnostic criteria such as CCMD-3. The specific criteria were as follows:

- P (Patients): Adults with insomnia disorders diagnosed according to standardized diagnostic criteria, including primary insomnia/chronic insomnia defined by DSM-IV, DSM-5, ICSD, ICD, or validated Chinese diagnostic criteria. Studies involving insomnia associated with specific medical conditions were considered eligible when insomnia was the primary treatment target and baseline insomnia symptoms were clearly defined.
- I (Interventions): Treatment involving TCM, including decoctions, granules, or other modern formulations, with or without pharmacological or psychotherapeutic interventions, for a minimum duration of 4 weeks. Studies were selected if they involved TCM used alone or as an adjunct to pharmacological interventions. Studies involving TCM combined with non-pharmacological methods (e.g., exercise, nutritional modifications, or other alternative therapies) were excluded. The minimum intervention duration was set at 4 weeks. Due to the limited number of eligible RCTs and substantial heterogeneity among individual herbal prescriptions, interventions were grouped according to clinically meaningful treatment categories. TCM-based interventions included different herbal formulas and patent medicines administered as the primary treatment. TCM combined with Western medicine referred to studies in which a TCM intervention was added to conventional pharmacotherapy. This categorization was predefined to ensure adequate network connectivity but may reduce the ability to distinguish effects among individual formulas.
- C (Controls): Comparators included pharmacological agents (e.g., benzodiazepines), psychotherapeutic interventions (e.g., cognitive behavioral therapy), placebo, or no treatment.
- (Outcomes): The primary outcomes included standardized sleep-related severity measures, specifically the Pittsburgh Sleep Quality Index (PSQI) and Insomnia Severity Index (ISI) scores. Secondary outcomes included clinical efficacy rate and adverse events (AEs). Clinical efficacy was analyzed as a dichotomous responder outcome based on the criteria reported in each original study. Clinical efficacy was defined according to the criteria reported in each original trial, which generally classified participants as responders when predefined symptom improvement thresholds were achieved, such as ≥30% reduction in PSQI score or investigator-defined improvement categories (e.g., cured, markedly effective, effective). Because different trials applied different response criteria, clinical efficacy was analyzed as reported and interpreted cautiously.
- S (Study Design): Prospective RCTs.

### Literature screening

Two researchers imported the retrieved articles into EndNote X7 for reference management and removed duplicates. Studies were screened based on their titles and abstracts to exclude obviously irrelevant articles. Irrelevant studies were excluded through abstract review, and full texts were examined to ensure final inclusion. The search and screening processes were conducted independently, with a third team member coordinating discrepancies and confirming the final list of included studies.

### Data extraction

Data from the selected studies were extracted independently by two reviewers using a standardized Excel template, including first author, publication year, participant characteristics, diagnostic criteria, intervention characteristics, comparator interventions, treatment duration, and outcome measures. For each outcome, the number of participants, event counts for dichotomous outcomes, and mean values with standard deviations for continuous outcomes were extracted.

When continuous outcomes were reported as medians and interquartile ranges (IQRs), means and standard deviations were estimated using the methods proposed by Wan et al. (2014) (18). Specifically, sample size, median, range, and IQR values were used to approximate the corresponding mean and standard deviation. When only median and IQR values were available, the standard deviation was estimated using the approximation SD ≈ IQR/1.35.

For continuous outcomes, post-treatment scores were preferentially extracted; when only change-from-baseline values were available, the reported change scores were used. Dichotomous outcomes, including clinical efficacy and adverse events, were analyzed using risk ratios (RRs) or odds ratios (ORs) with 95% confidence intervals (CIs). Multi-arm trials were handled using appropriate network meta-analysis methods to avoid double-counting of shared comparator groups. For studies with zero-event outcomes, continuity corrections were applied when necessary.

Missing or unclear information was addressed by contacting the corresponding authors when possible. If additional data could not be obtained, analyses were conducted based on available data, and the potential influence of missing information was considered during interpretation. Any discrepancies in data extraction were resolved through discussion between reviewers, with arbitration by a third reviewer when necessary.

### Geometry of the network

A network plot was drawn to represent the treatment relationships among the included studies. Each node represented a treatment strategy category rather than a specific herbal formula. The predefined nodes included TCM-based therapy alone, TCM combined with Western medicine, Western medicine alone, placebo, and CBT-I. Because individual TCM prescriptions are highly heterogeneous and are selected according to syndrome differentiation, direct interchangeability between specific formulas cannot be assumed. Therefore, this network meta-analysis aimed to compare broader treatment strategies rather than individual TCM formulations. The thickness of the edges corresponds to the number of trials between interventions. This network structure description adheres to the PRISMA-NMA standards for assessing network connectivity and transitivity.

### Risk of bias assessment

Two reviewers independently assessed the risk of bias using the Cochrane Risk of Bias tool (RoB 1.0) described in the Cochrane Handbook for Systematic Reviews of Interventions (Version 5.1.0). RoB 1.0 was selected because most included RCTs were published before widespread adoption of RoB 2.0 and frequently lacked sufficient reporting details required for the domain-based judgment framework of RoB 2.0. The assessment included seven domains: random sequence generation, allocation concealment, blinding of participants and personnel, blinding of outcome assessors, incomplete outcome data, selective reporting, and other bias.

Given the incomplete reporting of methodological details in several older RCTs, some domains were judged as unclear risk rather than high risk, reflecting insufficient information rather than confirmed methodological limitations.

### Assessment of transitivity and inconsistency

Transitivity was assessed by comparing potential effect modifiers across treatment contrasts, including participant age, sex distribution, insomnia diagnostic criteria, baseline disease characteristics, comorbid conditions, intervention duration, comparator type, and outcome definitions. Although clinical heterogeneity existed, these characteristics were considered sufficiently comparable because all studies enrolled adults with clinically significant insomnia symptoms and evaluated standardized sleep-related outcomes. The potential influence of secondary or comorbid insomnia populations was further examined through sensitivity analysis excluding these studies. A design-by-treatment interaction model was used to evaluate the global inconsistency of the network. The node-splitting technique was employed to compare direct and indirect evidence to examine local inconsistency. These analyses were conducted in accordance with PRISMA-NMA recommendations and were performed using Stata software.

### Statistical methods

This study employed a frequentist random-effects network meta-analysis model using Stata software. SUCRA values were calculated to summarize the probability ranking of each intervention, ranging from 0 to 100%. For efficacy outcomes (ISI, PSQI, and clinical efficacy rate), higher SUCRA values indicated a greater probability of achieving better clinical outcomes. For safety outcomes, adverse events were coded as undesirable events; therefore, the ranking direction was reversed, with higher SUCRA values representing a lower probability of adverse events and better safety.

Because SUCRA rankings alone do not provide information regarding effect magnitude, statistical uncertainty, or clinical relevance, all treatment comparisons were additionally presented using league tables and forest plots with relative effect estimates (odds ratios [ORs] or mean differences [MDs]) and corresponding 95% confidence intervals (CIs).

## Results

### Basic information of included studies

The flow chart of the study selection process was presented according to the PRISMA-NMA guidelines, including literature identification, screening, eligibility assessment, and final inclusion. Specific reasons for exclusion were provided for studies excluded during the full-text assessment. As illustrated in Figure 1, a total of 1,172 records were identified from electronic databases and other sources. These included 1,157 records from database searches (PubMed, Embase, Web of Science, Cochrane CENTRAL, ClinicalTrials.gov, CNKI, Wanfang Data, VIP Database, and SinoMed) and 15 additional records from other sources. After removal of 312 duplicate records, 860 records underwent title and abstract screening. During this stage, 774 records were excluded because they did not meet the predefined eligibility criteria. Subsequently, 86 full-text articles were assessed for eligibility, and 74 articles were excluded for the following reasons: intervention duration shorter than 4 weeks (*n* = 11), absence of relevant outcome data (*n* = 49), duplicate or overlapping populations (*n* = 4), and comparisons between different TCM formulations without an eligible control group (*n* = 10). Finally, 12 randomized controlled trials involving 1,260 participants were included in the network meta-analysis (19–30). Those 12 studies encompassed a total of 1,260 participants, consisting of 10 two-arm studies and 2 three-arm studies. Based on treatment strategies, the included studies were categorized into five network nodes: TCM-based therapy alone, TCM combined with Western medicine, Western medicine alone, placebo, and CBT-I. These categories represented broader therapeutic approaches rather than interchangeable individual herbal formulas. Commonly extracted outcome measures included the ISI, PSQI, clinical efficacy, and adverse events (AEs). To meet the PRISMA-NMA criteria, the study characteristics were assessed for clinical comparability, such as participant demographics, type of interventions, course of treatment, and timing of outcomes. These similarities validated the transitivity assumption necessary for conducting a network meta-analysis. Table 1 summarizes the basic data of the included studies.

**Figure 1:**
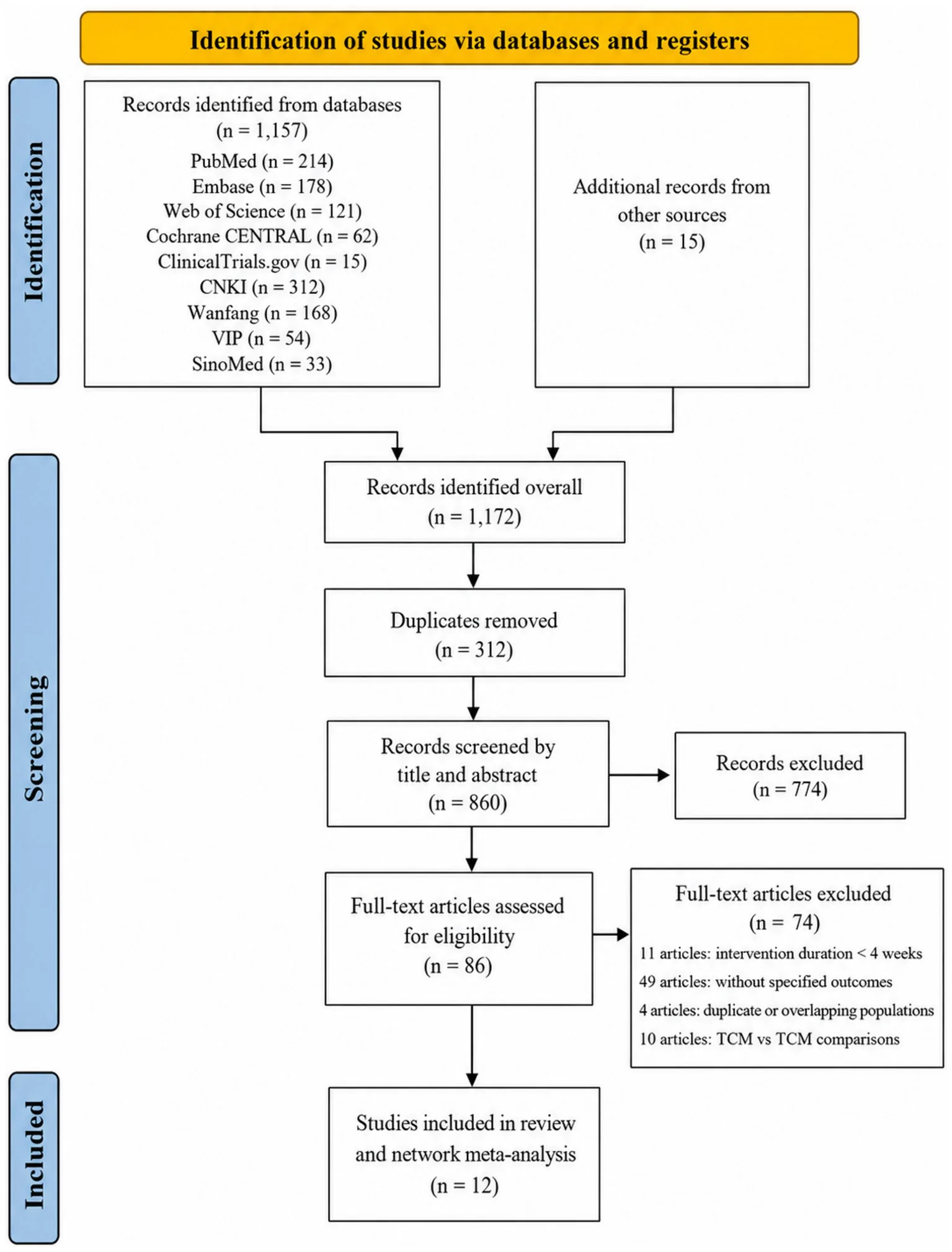
Literature screening process.

**Table 1:** The basic data of the included studies.

First author	Year	Interventions	Sample size	Follow-up time	Outcomes	Diagnostic criteria
Birling et al. (19)	2022	Zao Ren An Shen capsule vs. placebo	85	4 weeks	ISI, AEs	DSM-5 criteria for insomnia disorder / clinical diagnostic criteria
Zhu et al. (20)	2022	Zao Ren An Shen capsule + zolpidem vs. zolpidem	96	4 weeks	ISI, clinical efficacy, AEs	Chinese diagnostic criteria for insomnia (clinical diagnostic criteria)
Gan et al. (21)	2013	Zao Ren An Shen capsule vs. alprazolam	120	4 weeks	PSQI, clinical efficacy, AEs	Clinical diagnostic criteria for insomnia
Hu et al. (22)	2015	Suan Zao Ren Tang plus ZhiZiChiTang vs. lorazepam	128	4 weeks	ISI, PSQI	Insomnia with anxiety symptoms; diagnostic criteria for insomnia with comorbid anxiety
Song et al. (23)	2020	Jiawei Suanzaoren decoction plus lorazepam vs. lorazepam	207	4 weeks	ISI, AEs	Chronic insomnia (diagnostic criteria reported in original study; specific criteria not available)
Lin et al. (24)	2013	Wuling capsule vs. placebo	186	6 weeks	PSQI, AEs	Insomnia disorder (clinical diagnostic criteria; specific criteria not reported)
Dai et al. (25)	2020	Ningxin Anshen formula vs. zopiclone vs. placebo	90	4 weeks	PSQI, clinical efficacy, AEs	Post-stroke insomnia
Zhu et al. (26)	2023	Modified Suanzaoren decoction vs. zolpidem	80	4 weeks	PSQI, AEs	Post-stroke cognitive impairment with comorbid insomnia symptoms
Chan et al. (27)	2015	Suan Zao Ren Tang plus methadone vs. methadone	90	4 weeks	PSQI, AEs	Sleep disturbance during methadone maintenance treatment
Chen et al. (28)	2022	Shumian capsule vs. placebo	26	4 weeks	ISI, PSQI	Insomnia disorder (specific diagnostic criteria not reported)
Wang et al. (29)	2024	Zhumian Tang formula granules plus eszopiclone vs. eszopiclone	130	4 weeks	PSQI, clinical efficacy, AEs	Poor sleep quality/insomnia symptoms (specific diagnostic criteria not reported)
Moon et al. (30)	2022	Tian Wang Bu Xin Dan vs. cognitive behavioral therapy	22	4 weeks	ISI, PSQI	Cancer-related insomnia

Diagnostic criteria were extracted as reported in the original studies. When specific diagnostic criteria were not explicitly described, this was recorded as “not reported.” Studies involving secondary or comorbid insomnia populations (including post-stroke insomnia, anxiety-associated insomnia, methadone-maintenance-related sleep disturbance, and cancer-related insomnia) were retained because insomnia symptoms represented the primary treatment target and sleep-related outcomes were specifically evaluated.

Overall, most studies showed unclear risk in allocation concealment and selective reporting because methodological details were insufficiently reported. Blinding-related domains were frequently judged as unclear or high risk due to the nature of herbal interventions and comparator designs. No study was judged as having a high risk of bias across all domains.

The risk of bias assessment of the included studies is presented in Figure 2.

**Figure 2:**
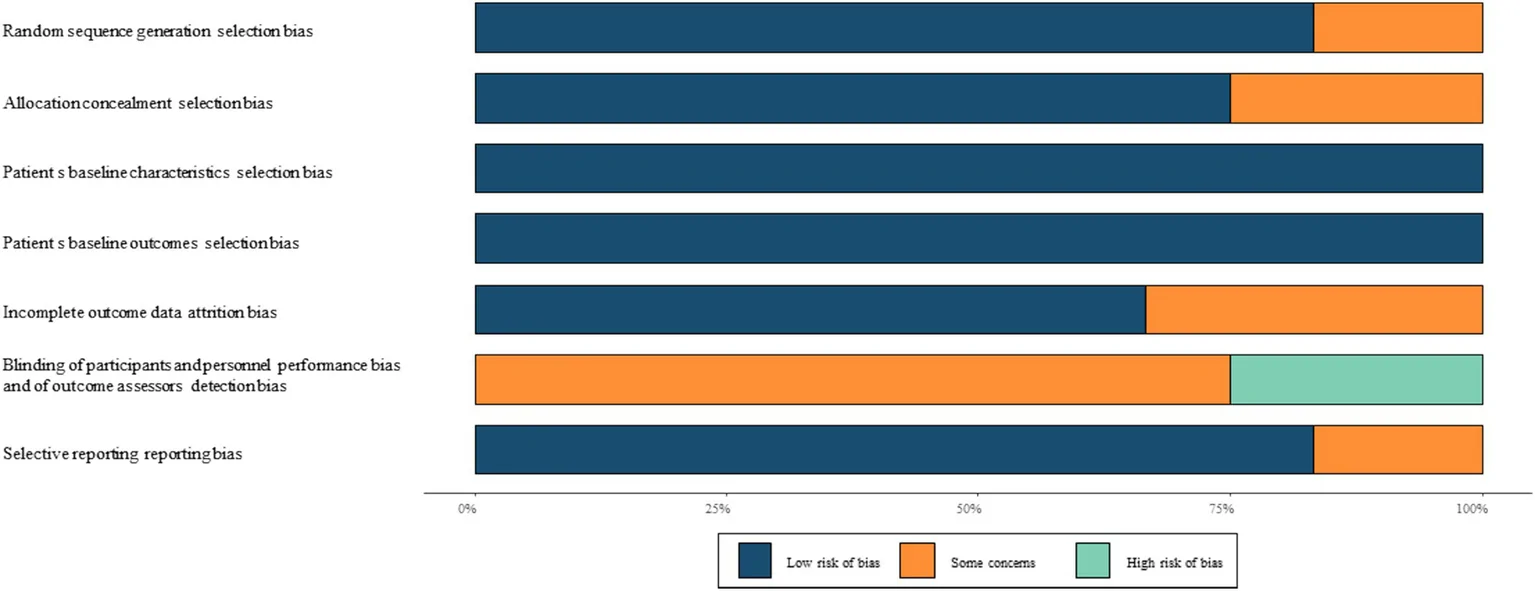
Risk of bias assessment of included randomized controlled trials using the Cochrane Risk of Bias tool (RoB 1.0).

### Network diagram

A network diagram was constructed to illustrate the quantity of studies and participants comparing different treatment modalities. For both ISI and PSQI outcomes, the majority of studies concentrated on comparisons between TCM and Western medicine, as well as between TCM and Placebo. Only one study reported on the comparison between CBT-I and TCM. In terms of clinical efficacy, most studies compared TCM and Western medicine, followed by comparisons between TCM plus Western medicine and Western medicine alone. Regarding adverse events, research primarily focused on comparisons between TCM and Placebo, as well as between Western medicine and TCM plus Western medicine. The network geometry was checked, as per PRISMA-NMA requirements, to warrant sufficient connection and allow for indirect comparisons. The network revealed that the majority of interventions were well-connected, enabling valid synthesis. The network diagram is presented in Figure 3.

**Figure 3:**
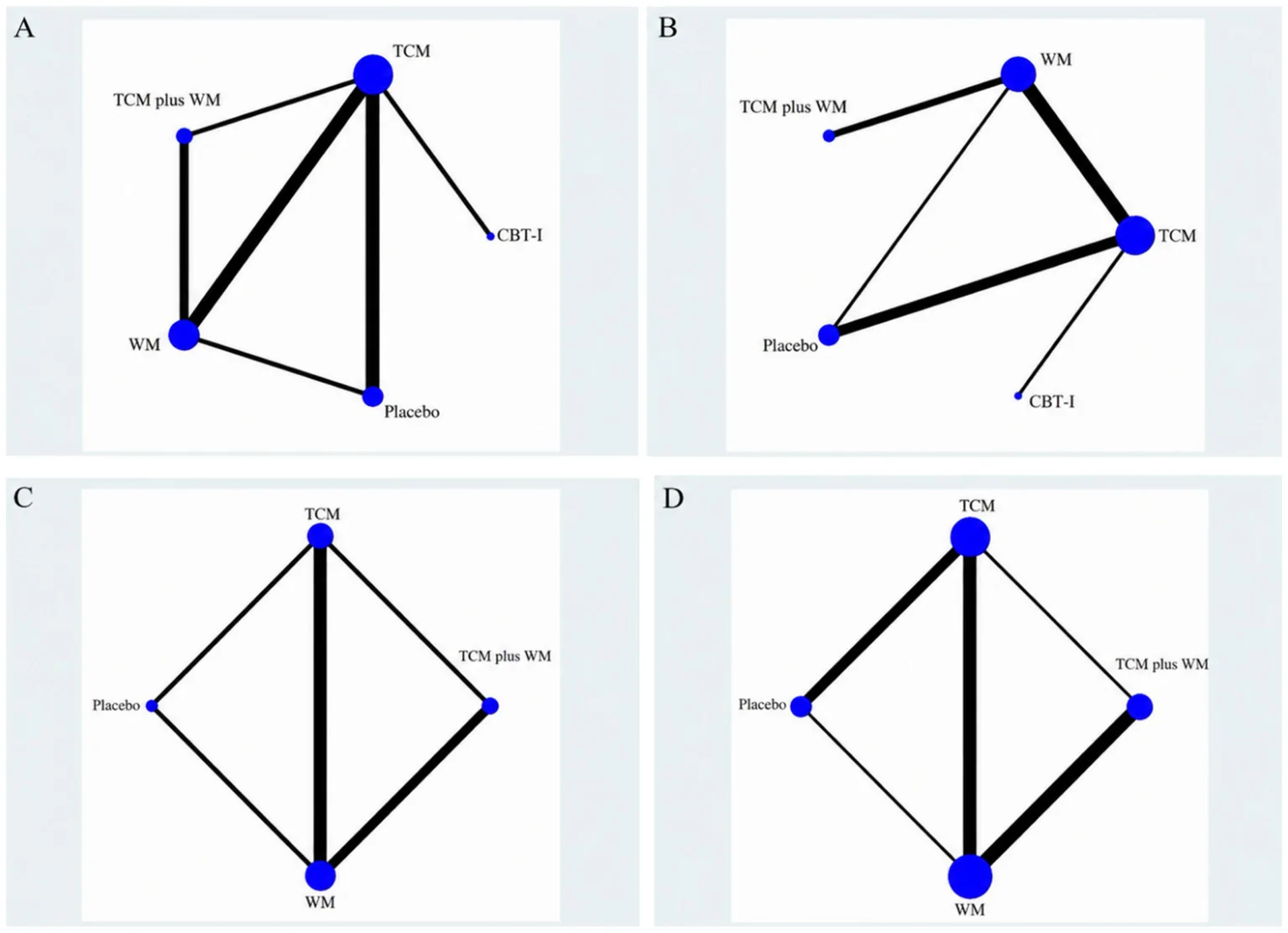
Network plots of treatment comparisons for insomnia outcomes. **(A)** Insomnia Severity Index (ISI); **(B)** Pittsburgh Sleep Quality Index (PSQI); **(C)** Clinical efficacy; **(D)** Adverse events (AEs). The size of each node represents the total number of participants receiving the corresponding intervention, and the thickness of each connecting line represents the number of direct comparisons between interventions. TCM, Traditional Chinese Medicine; WM, Western medicine.

Global inconsistency was assessed using the design-by-treatment interaction model. No statistically significant inconsistency was detected for ISI outcomes (χ^2^ = 4.21, *p* = 0.518), PSQI outcomes (χ^2^ = 5.37, *p* = 0.497), clinical efficacy outcomes (χ^2^ = 2.16, *p* = 0.539), or adverse event outcomes (χ^2^ = 1.84, *p* = 0.606), suggesting acceptable agreement between direct and indirect evidence.

Local inconsistency was evaluated using node-splitting analysis. No significant disagreement between direct and indirect estimates was observed for the available comparisons (all *p* > 0.05). Comparisons involving TCM plus Western medicine versus Western medicine showed consistent direct and indirect estimates for ISI (*p* = 0.423) and PSQI (*p* = 0.378).

### Comparison of ISI outcomes across different treatments

Among the included studies, 7 reported ISI outcomes after treatment, comprising 2 three-arm studies and 5 two-arm studies, with treatment modalities including TCM, TCM plus Western medicine, Western medicine, Placebo, and CBT-I.

SUCRA values were calculated to describe the relative ranking probabilities of interventions. The ranking probabilities for ISI improvement were highest for TCM plus Western medicine (SUCRA 89.7%), followed by TCM alone (72.5%), WM alone (50.1%), CBT-I (19.9%), and placebo (17.9%).

However, SUCRA rankings were not considered evidence of statistical superiority. Relative treatment effects were additionally evaluated using network estimates. Compared with placebo, TCM plus Western medicine significantly improved ISI outcomes (OR 2.84, 95% CI 1.42–5.68). Compared with Western medicine alone, the difference was also favorable but with wider uncertainty (OR 1.96, 95% CI 1.08–3.56). Based on global and local inconsistency tests, no significant discrepancy was detected between the direct and indirect comparisons for the ISI outcomes. The SUCRA values for the ISI outcomes are presented in Figure 4.

**Figure 4:**
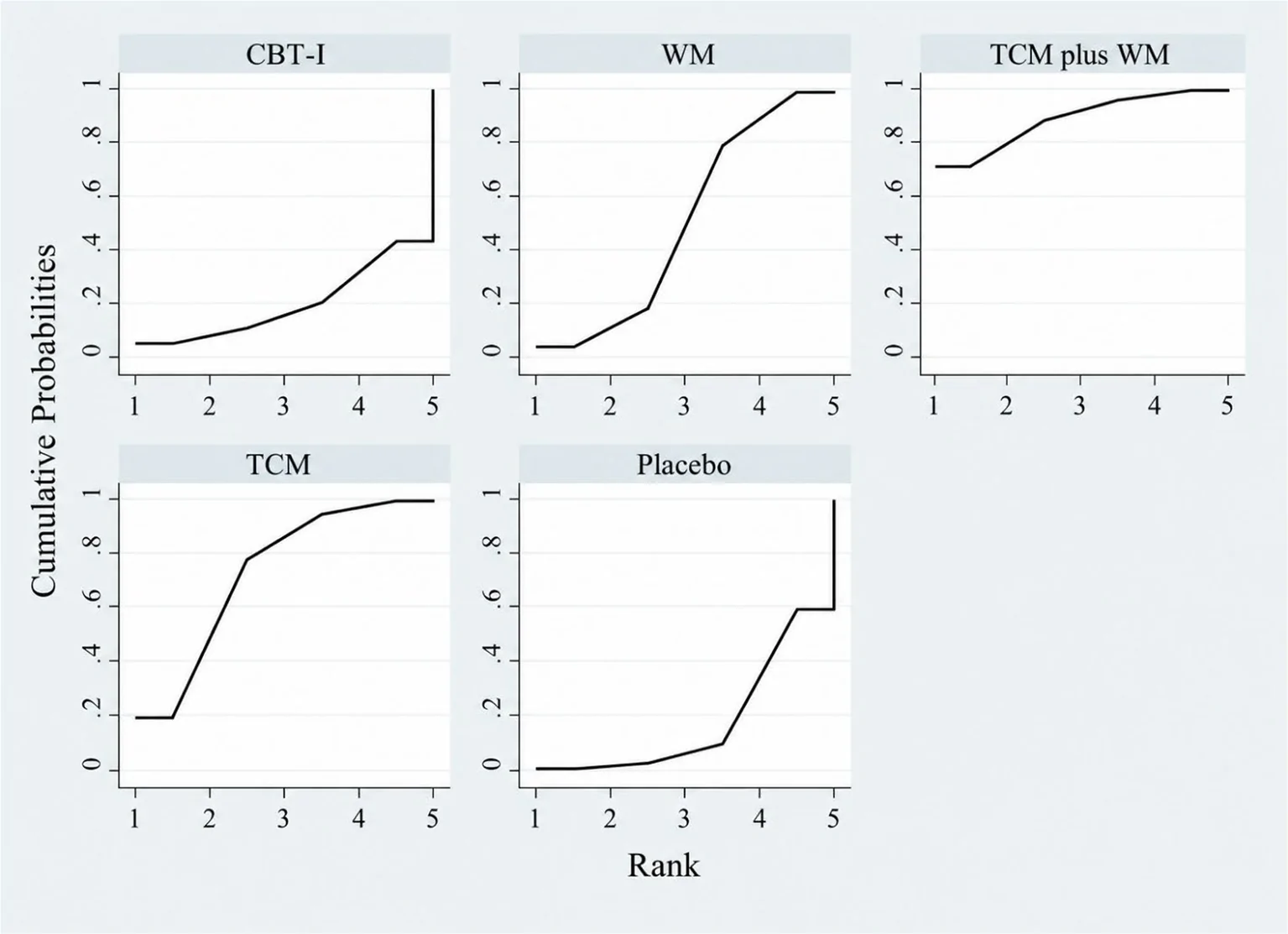
SUCRA ranking probabilities for ISI outcomes across predefined treatment categories. The SUCRA values represent the cumulative probability of each treatment strategy achieving different ranks for ISI improvement and should be interpreted together with comparative effect estimates and uncertainty intervals. TCM, Traditional Chinese Medicine; WM, Western medicine.

### Comparison of PSQI outcomes across different treatments

Nine studies reported PSQI outcomes after treatment, including one three-arm study and eight two-arm studies, with treatment modalities comprising TCM, TCM plus Western medicine, Western medicine, Placebo, and CBT-I. SUCRA values for each intervention indicated the following rankings: TCM plus Western medicine (71.9%), TCM (64.9%), Western medicine (49.8%), Placebo (40.8%), and CBT-I (22.7%). Statistical heterogeneity for the PSQI network was assessed using the random-effects model. No substantial statistical heterogeneity was observed among the included comparisons (τ^2^ = 0.08, I^2^ = 18.6%). However, considerable clinical heterogeneity existed across studies regarding insomnia populations, diagnostic criteria, intervention types, and treatment contexts. The cumulative ranking probabilities and corresponding SUCRA values for PSQI outcomes are presented in Figure 5.

**Figure 5:**
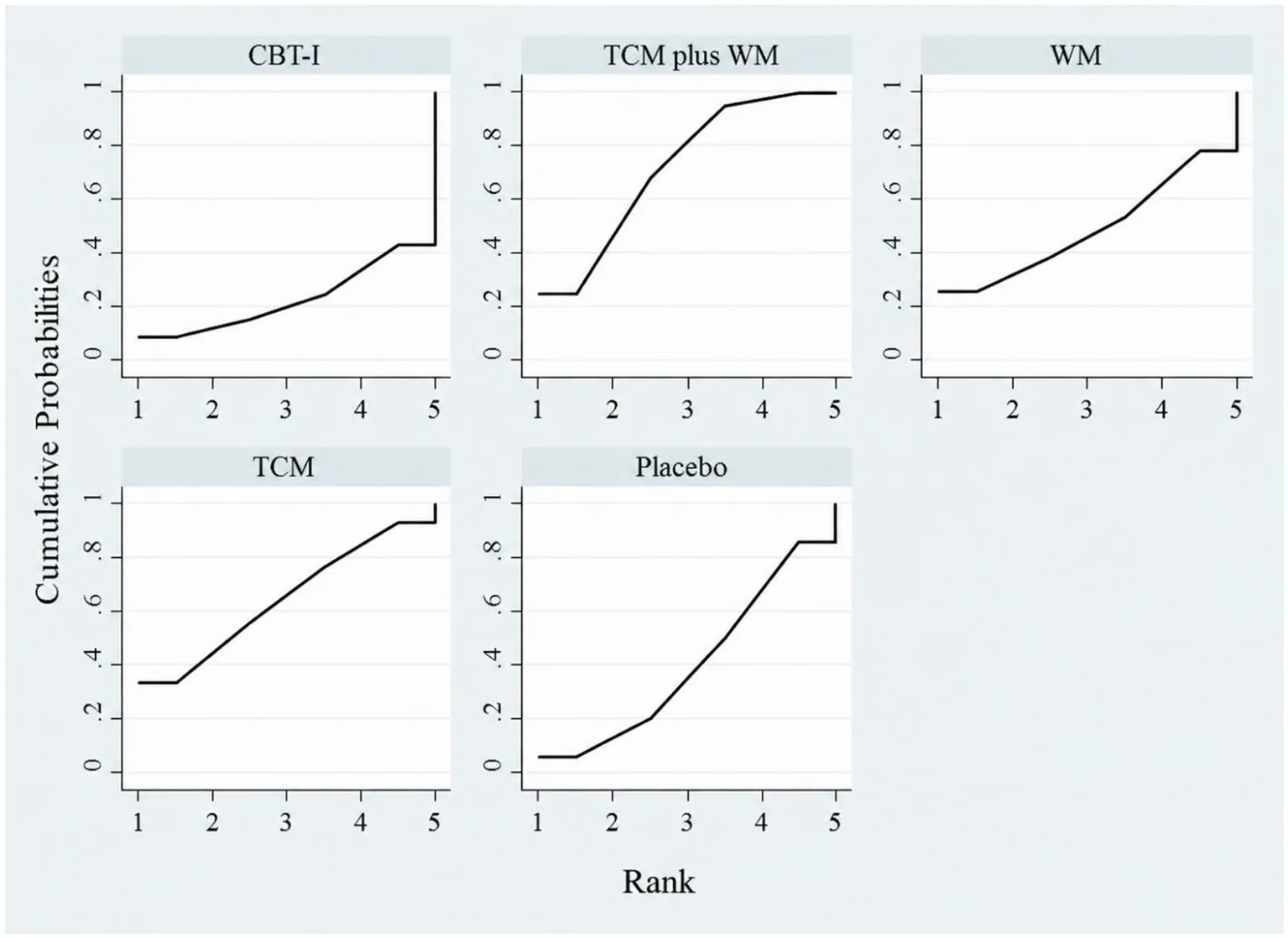
SUCRA ranking probabilities for PSQI outcomes across predefined treatment categories. The x-axis represents the rank of each intervention, and the y-axis represents cumulative ranking probabilities. Higher SUCRA values indicate a greater probability of achieving a favorable rank for PSQI improvement but do not necessarily indicate statistically significant superiority. TCM, Traditional Chinese Medicine; WM, Western medicine.

### Comparison of clinical efficacy across different treatments

Four studies provided data on clinical efficacy. Because response criteria varied among studies, clinical efficacy was analyzed according to each trial’s predefined responder definition. Most studies classified participants based on symptom improvement categories (including cured, markedly effective, effective, and ineffective), which were converted into dichotomous outcomes for network meta-analysis. The SUCRA ranking showed TCM plus Western medicine as the highest probability treatment (86.6%), followed by TCM (77.2%), Western medicine (20.9%), and placebo (15.3%). These rankings should be interpreted cautiously because the underlying response definitions were not completely identical across studies. The cumulative ranking probabilities and corresponding SUCRA values for clinical efficacy are presented in Figure 6.

**Figure 6:**
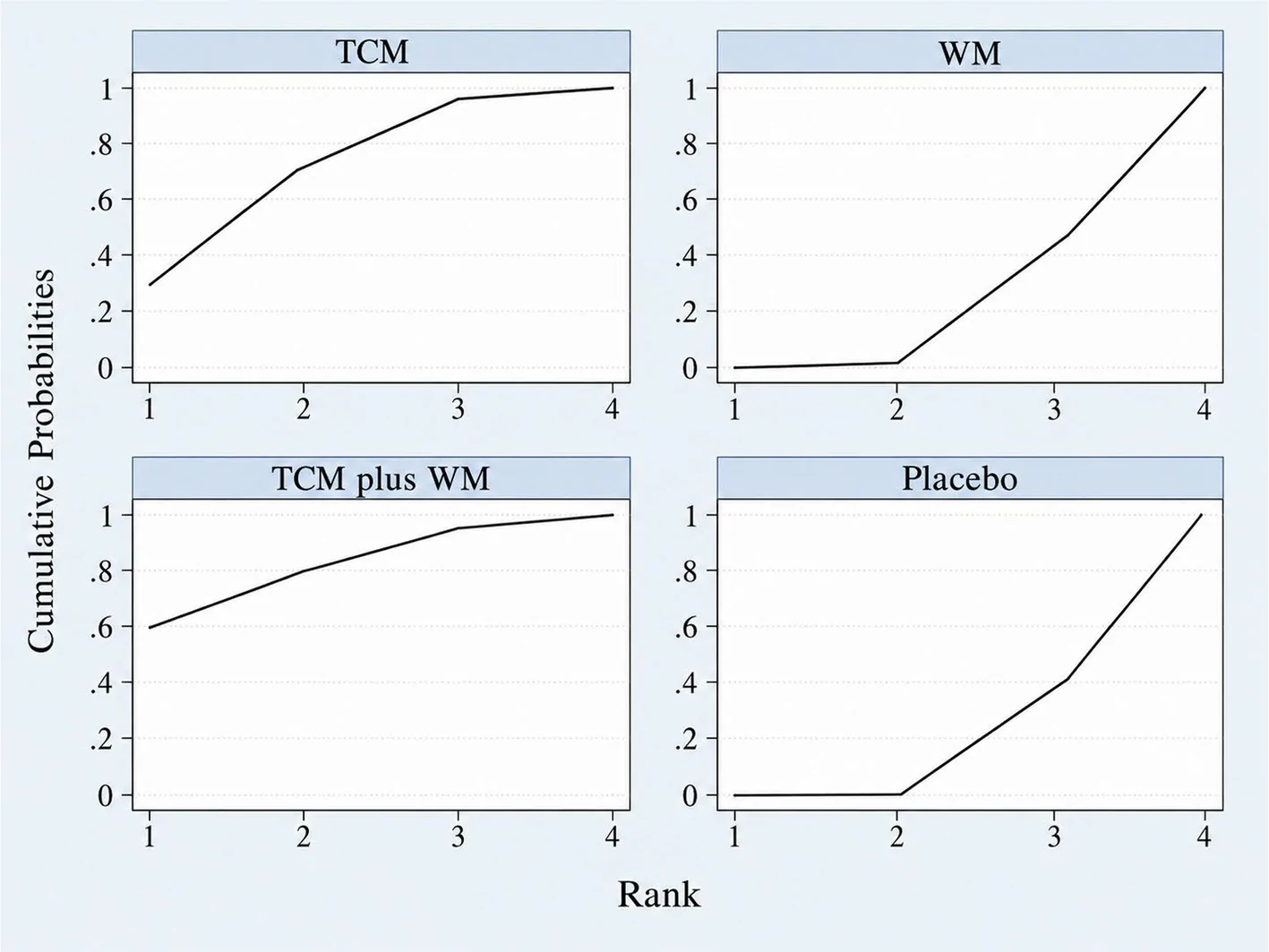
SUCRA ranking probabilities for clinical efficacy across predefined treatment categories. The x-axis represents intervention rank, and the y-axis represents cumulative ranking probabilities. Higher SUCRA values indicate a greater probability of achieving a favorable rank for clinical efficacy but do not necessarily indicate statistically significant superiority. TCM, Traditional Chinese Medicine; WM, Western medicine.

### Comparison of adverse event rates across different treatments

Seven studies reported on adverse event outcomes after treatment, including two three-arm studies and five two-arm studies, with treatment modalities comprising TCM, TCM plus Western medicine, Western medicine, and Placebo. For adverse events, the ranking direction was predefined such that higher SUCRA values represented a lower probability of experiencing adverse events. Therefore, SUCRA values were interpreted inversely compared with efficacy outcomes. The ranking probabilities were Western medicine (94.8%), TCM plus Western medicine (43.9%), placebo (31.1%), and TCM (30.2%).

Nevertheless, these rankings should not be interpreted independently. Compared with Western medicine alone, TCM combined with Western medicine was associated with a significantly lower risk of adverse events (OR 0.49, 95% CI 0.26--0.94), suggesting that the addition of TCM may mitigate the adverse effects of conventional pharmacotherapy. Conversely, Western medicine alone was associated with a significantly higher risk of adverse events compared with TCM alone (OR 2.41, 95% CI 1.12--5.18) and placebo (OR 2.06, 95% CI 1.01--4.20). The consistency test indicated good agreement between direct and indirect comparisons for the adverse event outcomes, meeting the methodological requirements of the PRISMA-NMA guidelines. The SUCRA values for the incidence of adverse events are presented in Figure 7.

**Figure 7:**
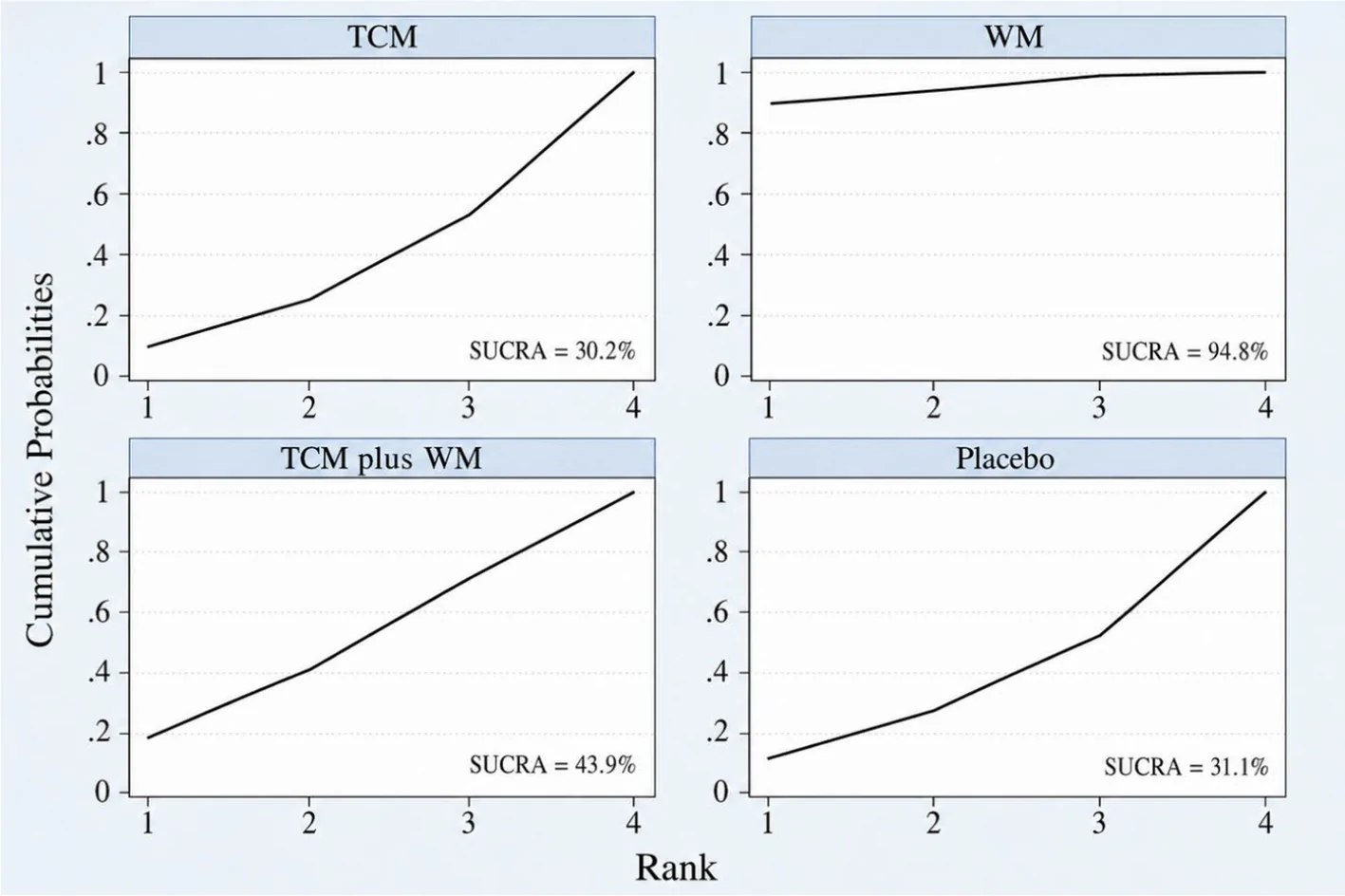
SUCRA ranking probabilities for adverse event outcomes across predefined treatment categories. For safety outcomes, higher SUCRA values indicate a greater probability of being ranked as having fewer adverse events. However, because the league table (Supplementary Table S4) provides direct comparative effect estimates with 95% confidence intervals, safety conclusions in this study are drawn primarily from these pairwise estimates rather than from SUCRA rankings alone. The figure is presented for descriptive purposes to illustrate ranking uncertainty.

To further quantify comparative treatment effects, network league tables and forest plots were generated for all evaluated outcomes, including ISI, PSQI, clinical efficacy, and adverse events. These analyses incorporated both direct and indirect comparisons and presented relative treatment effects with corresponding 95% confidence intervals, allowing assessment of effect magnitude and statistical uncertainty beyond treatment rankings. The league tables are provided in Supplementary Tables S1–S4.

Sensitivity analysis excluding studies involving secondary or comorbid insomnia populations (including post-stroke insomnia, cancer-related insomnia, anxiety-associated insomnia, and methadone-maintenance-related sleep disturbance) was performed to assess the robustness of the findings. After exclusion of these studies, eight RCTs involving 1,012 participants remained. The ranking patterns remained generally consistent, with TCM plus Western medicine maintaining the highest probability ranking for ISI improvement (SUCRA 87.5%) and PSQI improvement (SUCRA 69.8%). The direction and magnitude of treatment effects were comparable with the primary analysis, suggesting that inclusion of secondary insomnia populations did not substantially influence the overall conclusions.

## Discussion

It has been utilized for the treatment of insomnia for over two millennia, with its theoretical foundation rooted in regulating the balance of yin and yang among the body’s organs (31). The selection of specific herbal formulas depends on the underlying cause, pathophysiology, stage, phenotype, and accompanying physical disorders of insomnia. Commonly prescribed formulas include Wendan Tang, Guipi Tang, and Suanzaoren Tang (11). Although the TCM diagnostic and treatment system often employs syndrome differentiation and treatment, prescriptions based on syndromes can vary significantly among different practitioners. Methodological limitations in existing studies indicate that, while some research suggests TCM may be more effective and safer than conventional therapies, more rigorous investigations are still required (32, 33).

This study systematically evaluated the comparative efficacy and safety of different TCM-based treatment strategies for insomnia using a network meta-analysis framework. The results suggested that TCM-based therapies, particularly TCM combined with Western medicine, were associated with favorable improvements in insomnia-related outcomes compared with several control interventions. However, because network rankings are probability-based and influenced by evidence uncertainty, SUCRA values should not be interpreted as definitive evidence of treatment superiority. The combination of TCM and Western medicine showed the highest probability rankings for ISI (89.7%), PSQI (71.9%), and clinical efficacy (86.6%); nevertheless, these findings should be interpreted together with comparative effect estimates, confidence intervals, and clinical relevance. The risk of adverse events associated with TCM plus Western medicine was significantly lower than that of Western medicine alone (OR 0.49, 95% CI 0.26--0.94), suggesting a potential safety benefit of combined therapy. However, this finding should be interpreted cautiously due to the small number of studies and the heterogeneous nature of adverse event reporting across trials.

Importantly, the treatment nodes in this network meta-analysis represent broader therapeutic categories rather than individual herbal prescriptions. Although this approach improved network connectivity and enabled indirect comparisons, different TCM formulas may vary substantially in their pharmacological components, theoretical foundations, and clinical indications. Therefore, the findings should not be interpreted as demonstrating superiority of any specific herbal formula. Future well-designed trials directly comparing individual TCM formulations are warranted.

Previous studies have reported potential benefits of TCM-based interventions for insomnia; however, differences in study design, intervention classification, and methodological quality limit direct comparisons with the present network analysis (34, 35). It has been reported that the average effectiveness of TCM in different TCM patterns was between 75.0–98.1% (33). However, the general overall quality of research on TCM for insomnia is low (34, 36). A systematic review of 217 studies found that only eight studies had a score ≥3, indicating generally poor methodological quality; most RCTs mentioned randomization but did not provide further details on methodology, implementation of blinding, or dropout rates. These limitations hinder the drawing of definitive conclusions regarding efficacy (33).

This study employed relatively stringent literature inclusion criteria, thereby yielding more valid conclusions. TCM emphasizes individualized treatment based on syndrome differentiation, which can serve as a complement to standardized Western medical practice and may improve overall treatment outcomes (31). Studies have also indicated that certain TCM possess sedative effects, with mechanisms involving the modulation of serotonin 1A receptors or action via the neurotransmitter gamma-aminobutyric acid (GABA), suggesting potential synergistic effects when combined with Western pharmaceuticals (37). Currently, the existing literature is primarily based on comparisons with a vehicle or Western medicine, while studies observing the combination of TCM and Western medicine for insomnia are relatively scarce. One of the main challenges in treating insomnia is drug tolerance (38), which provides a rationale for the belief that TCM may have significant long-term positive impacts, although corresponding research remains limited. Future research directions could include employing more strategies to evaluate the efficacy of TCM for insomnia, such as conducting large-scale, multicenter RCTs, longitudinal observational studies, pharmacological mechanistic studies, or research on combination therapy with other complementary therapies. These strategies will be used to support and extend the findings of the current network meta-analysis.

However, this study also has several limitations. First, although statistical heterogeneity was generally limited in the network analyses, considerable clinical heterogeneity existed among included studies because insomnia populations varied, including primary insomnia and insomnia associated with neurological, psychiatric, or oncological conditions. Differences in diagnostic criteria, intervention characteristics, and treatment duration may influence treatment effects. Second, despite the implementation of strict inclusion criteria, issues remained regarding informed consent, proper implementation of blinding, and allocation concealment. Third, although both English and Chinese databases were searched to improve evidence coverage, most included studies were conducted in China, which may limit the generalizability of the findings to other healthcare systems. Future multicenter international trials with standardized diagnostic criteria are needed. Therefore, higher-level clinical studies are still needed to comprehensively evaluate the efficacy of TCM in the treatment of insomnia.

## Conclusion

This study provides comparative evidence regarding different TCM-based treatment strategies for insomnia. TCM combined with Western medicine showed favorable probability rankings for several outcomes; however, these findings should be interpreted cautiously because SUCRA rankings represent relative probabilities rather than definitive treatment effects. Furthermore, network nodes represented treatment categories rather than individual herbal prescriptions, and future high-quality RCTs directly comparing specific formulations are needed.

## Data Availability

The raw data supporting the conclusions of this article will be made available by the authors, without undue reservation.
