# Supplementary material for: Comparative effectiveness of traditional Chinese medicine treatment strategies for insomnia: a systematic review and network meta-analysis
Source: Front Neurol. 2026 Sep 25;17:1912776. doi: 10.3389/fneur.2026.1912776 (PMC13649412; doi:10.3389/fneur.2026.1912776)
Supplement: Supplementary file 1 [file Table_1.DOCX]

# Supplementary Material. Complete search strategies for all electronic databases

| PubMed  **Search date:** From inception to January 31, 2026 | #1  ("Insomnia"[Mesh]  OR insomnia  OR insomnia disorder  OR chronic insomnia  OR sleep disturbance  OR sleep initiation dysfunction  OR sleep quality  OR sleeplessness)  AND  #2  ("Medicine, Chinese Traditional"[Mesh]  OR Traditional Chinese Medicine  OR Chinese Medicine  OR Chinese herbal medicine  OR herbal formula  OR herbal medicine  OR TCM  OR TCM formulation)  AND  #3  (randomized controlled trial  OR randomized  OR randomised  OR clinical trial  OR controlled trial  OR random allocation) |
| --- | --- |
| Embase  **Search date:** From inception to January 31, 2026 | #1  ('insomnia'/exp  OR insomnia  OR 'insomnia disorder'  OR 'chronic insomnia'  OR 'sleep disturbance'  OR 'sleep initiation dysfunction'  OR 'sleep quality')  AND  #2  ('traditional Chinese medicine'/exp  OR 'Chinese medicine'  OR 'Chinese herbal medicine'  OR herbal formula  OR TCM  OR 'TCM formulation')  AND  #3  ('randomized controlled trial'/exp  OR random*  OR 'clinical trial'  OR 'controlled clinical trial') |
| Web of Science Core Collection  **Search date:** From inception to January 31, 2026 | TS=  (insomnia  OR "insomnia disorder"  OR "chronic insomnia"  OR "sleep disturbance"  OR "sleep initiation dysfunction"  OR "sleep quality")  AND  TS=  ("Traditional Chinese Medicine"  OR "Chinese Medicine"  OR "Chinese herbal medicine"  OR "herbal formula"  OR TCM  OR "TCM formulation")  AND  TS=  (random*  OR "randomized controlled trial"  OR "clinical trial"  OR "controlled trial") |
| Cochrane CENTRAL  **Search date:** From inception to January 31, 2026 | #1  (insomnia OR "insomnia disorder"  OR "chronic insomnia"  OR "sleep disturbance")  AND  #2  ("Traditional Chinese Medicine"  OR "Chinese herbal medicine"  OR herbal medicine  OR TCM)  AND  #3  (randomized controlled trial  OR random*  OR clinical trial) |
| ClinicalTrials.gov  **Search date:** From inception to January 31, 2026 | Condition/Disease:  (insomnia OR sleep disorder OR sleep disturbance)  AND  Other terms:  (Traditional Chinese Medicine  OR Chinese herbal medicine  OR TCM)  Study type:  Interventional Studies  +  Randomized allocation |
| CNKI (中国知网)  **检索时间：建库至2026年1月31日** | 主题:  (失眠 OR 失眠症 OR 睡眠障碍 OR 慢性失眠 OR 睡眠质量下降)  AND  (中医 OR 中药 OR 中药复方 OR 中成药 OR 中医药)  AND  (随机 OR 随机对照试验 OR 临床试验 OR 对照试验) |
| Wanfang Data（万方数据库）  **检索时间：建库至2026年1月31日** | 主题:  (失眠 OR 失眠症 OR 睡眠障碍 OR 慢性失眠)  AND  (中医 OR 中药 OR 中药复方 OR 中成药)  AND  (随机对照试验 OR 随机 OR 临床试验) |
| VIP Database（维普数据库）  **检索时间：建库至2026年1月31日** | 主题:  (失眠 OR 睡眠障碍 OR 慢性失眠)  AND  (中医 OR 中药 OR 中药复方 OR 中成药)  AND  (随机 OR 随机对照试验 OR 临床试验) |
| SinoMed（中国生物医学文献数据库）  **检索时间：建库至2026年1月31日** | (失眠 OR 睡眠障碍 OR 慢性失眠)  AND  (中医 OR 中药 OR 中药复方 OR 中成药)  AND  (随机对照试验 OR 随机 OR 临床试验) |

Abbreviations: TCM, Traditional Chinese Medicine; CENTRAL, Cochrane Central Register of Controlled Trials. The search strategies were adapted according to the indexing systems and interfaces of each database. No language restrictions were applied. The final search was conducted on January 31, 2026. Both English and Chinese databases were searched to minimize language and publication bias.
